# Does agricultural cooperative membership impact technical efficiency of maize production in Nigeria: An analysis correcting for biases from observed and unobserved attributes

**DOI:** 10.1371/journal.pone.0245426

**Published:** 2021-01-22

**Authors:** Kehinde Oluseyi Olagunju, Adebayo Isaiah Ogunniyi, Zainab Oyetunde-Usman, Abiodun Olusola Omotayo, Bola Amoke Awotide

**Affiliations:** 1 Economics Research Branch, Agri-Food and Biosciences Institute, Belfast, United Kingdom; 2 International Food Policy Research Institute (IFPRI), Abuja, Nigeria; 3 Natural Resources Institute, University of Greenwich, Chatham Maritime, United Kingdom; 4 Food Security and Safety Niche Area, Faculty of Natural and Agricultural Sciences, North West University, Mmabatho, South Africa; 5 International Institute of Tropical Agriculture (IITA), Bamako, Mali; Universidad de Murcia, SPAIN

## Abstract

The formation of agricultural cooperatives has been widely promoted as an agricultural development policy initiative to help smallholder farmers cope with multiple production and marketing challenges. Using a nationally representative survey dataset of smallholder maize producers from rural Nigeria, this study assesses the impact of agricultural cooperative membership on technical efficiency (TE). We based our estimation approach on the combination of a newly developed sample selection stochastic production frontier model with propensity score matching to control for possible selectivity biases from both observables and unobservables. We estimate stochastic meta-frontiers to examine TE differences between cooperative members and non-members. Our results reveal that TE levels of members are consistently higher than that of non-members. This calls for continued policy incentives targeted at encouraging farmers to form as well as participate in agricultural cooperatives.

## 1. Introduction

The majority of rural households in Nigeria depend on agriculture as their main source of income. In 2018, agriculture generates approximately 70% of total rural jobs, accounts for more than 85% of rural income streams, and contributes about 25% of the country’s total GDP [1]. The sector is mainly dominated by small scale farmers who face multiple marketing and productivity challenges including limited access to productive inputs, output markets, extension services, credit facilities, and unavailability of improved agricultural technologies. This may lead to a reduction in agricultural incomes as well as constitute threats to the food security of rural households. The continued challenges faced by these farmers spurred several development parastatals, agribusiness companies, governments, and international development agencies to encourage farmers to form agricultural cooperatives as a policy initiative to enhance agricultural development as well as value chain development in transition and developing economies.

There is well-documented empirical evidence on the roles of agricultural cooperatives in enhancing the adoption of improved agricultural technologies and land management practices [2–8], economic performance and welfare of smallholder farmers [4, 9–15]. For instance, the study conducted in rural Nigeria by Wossen et al. [4] established the positive impacts of cooperatives on improved agricultural technology adoption and household welfare. Similarly, Michalek et al. [10] showed that farmers that belong to producer organisations have higher value-added, profitability, labour productivity, and employment than non-members. Membership of cooperatives is not only viable in increasing productive inputs use but also maximises farm outcomes by facilitating efficient use of inputs [16].

Despite growing interest from policymakers on the important roles of agricultural cooperatives in improving technical efficiency (TE) and yield of smallholder farmers especially in developing countries, only a few empirical studies have attempted to examine this important subject [3, 16–18]. Interestingly, the results from these few studies have been mixed. For example, studies such as Abate et al. [17] for Ethiopia, Ainembabazi et al. [3] for the Great Lake region of Africa, and Gedara et al. [19] for Sri Lanka reported a positive impact of agricultural cooperatives on productive efficiency. Conversely, Hailu et al. [20] and Wollni and Brümmer [21] reported an insignificant impact for teff farmers and coffee farmers in Ethiopia and Costa Rica, respectively. Possible reasons for this mixed evidence include differences in the structure of the formation and operation of cooperatives, and estimation techniques employed. Hence, the unclear empirical relationship between cooperative membership and TE requires further re-examination.

The present study aims at investigating the impact of agricultural cooperative membership on TE of maize production in rural Nigeria. Our paper makes important contributions to the literature. Firstly, we provide the first attempt, in the Nigeria context, to examine the impact of agricultural cooperatives on TE of maize farmers while making sure that we corrected for potential sources of selection biases from unobserved attributes by employing a selectivity-corrected stochastic frontier technique developed by Greene [22] and propensity score matching approach to correct for the selection bias stemming from observable factors. Indeed, previous studies on this subject, for example, Hailu et al. [20], Ainembabazi et al. [3], and Wollni and Brümmer [21] assume that the decision to participate in cooperatives or otherwise is random and may not be affected by unobserved factors. A few studies related to this subject in Nigeria, to our utmost knowledge, also follow suit on this assumption and consequently included cooperative membership as an explanatory variable in the efficiency model (for example [23, 24]). However, in reality, farmer’s decision to join cooperatives is non-random which simply implies that such a decision is based on the individual farmer’s self-selection into membership. Therefore, the duo of unobserved factors (such as risk behaviour and farmer managerial skills) and observed factors (such as household size, literacy level, and age) may affect farmer’s decision to participate which may lead to sample selection bias. It is therefore important to correct for these biases in order to ensure that the results obtained are unbiased, consistent, and suitable for policy recommendation. Secondly, unlike studies by Ma et al. [16] and Abdul-Rahaman and Abdulai [18], we estimate stochastic meta-frontiers to produce a common technology required for direct TE comparison between cooperative members and non-members, and further makes it possible to disentagle the impact of cooperative membership on technoloy gap and farm managerial ability. Finally, we assess the farm managerial, socioeconomic and plot-specific factors affecting the decision of maize farmers to participate in agricultural cooperatives, using a comprehensive survey data that cuts across the six geopolitical zones of Nigeria. This is key for formulating effective policy that will help unmask the constraints and incentives related with farmers participation in cooperatives in rural Nigeria, sub-Saharan Africa and other parts of the world as a whole.

The rest of the article is structured as follows: Section 2 provides background information as well as related literature on the roles of agricultural cooperatives. Section 3 outlines the conceptual and analytical frameworks for the study. This is followed by the presentation of data and descriptive statistics of all the variables used. Subsequently, the results and discussion section is presented in Section 5. Finally, we conclude the paper in section 6.

## 2. Context and related literature

The focus of this study is maize production in Nigeria, the largest producer of maize in Africa. It is one of the most grown cereal crops with the highest number of growers and the largest acreage under cultivation [25]. Between the period 2003–2011, the mean yearly acreage under maize production was 3 million hectares, accounting for approximately 23% of the total land area used for cereal crop production in the country [26]. Besides, maize is an important traditional food staple making up about 55% of the daily diets of rural and urban households in the country [27], thereby providing insurance against hunger. In the last two decades, its increasing multiple uses makes it vital not only as a food crop but also as a cash crop, thereby providing income sources for rural farming households [28].

**Fig 1:**
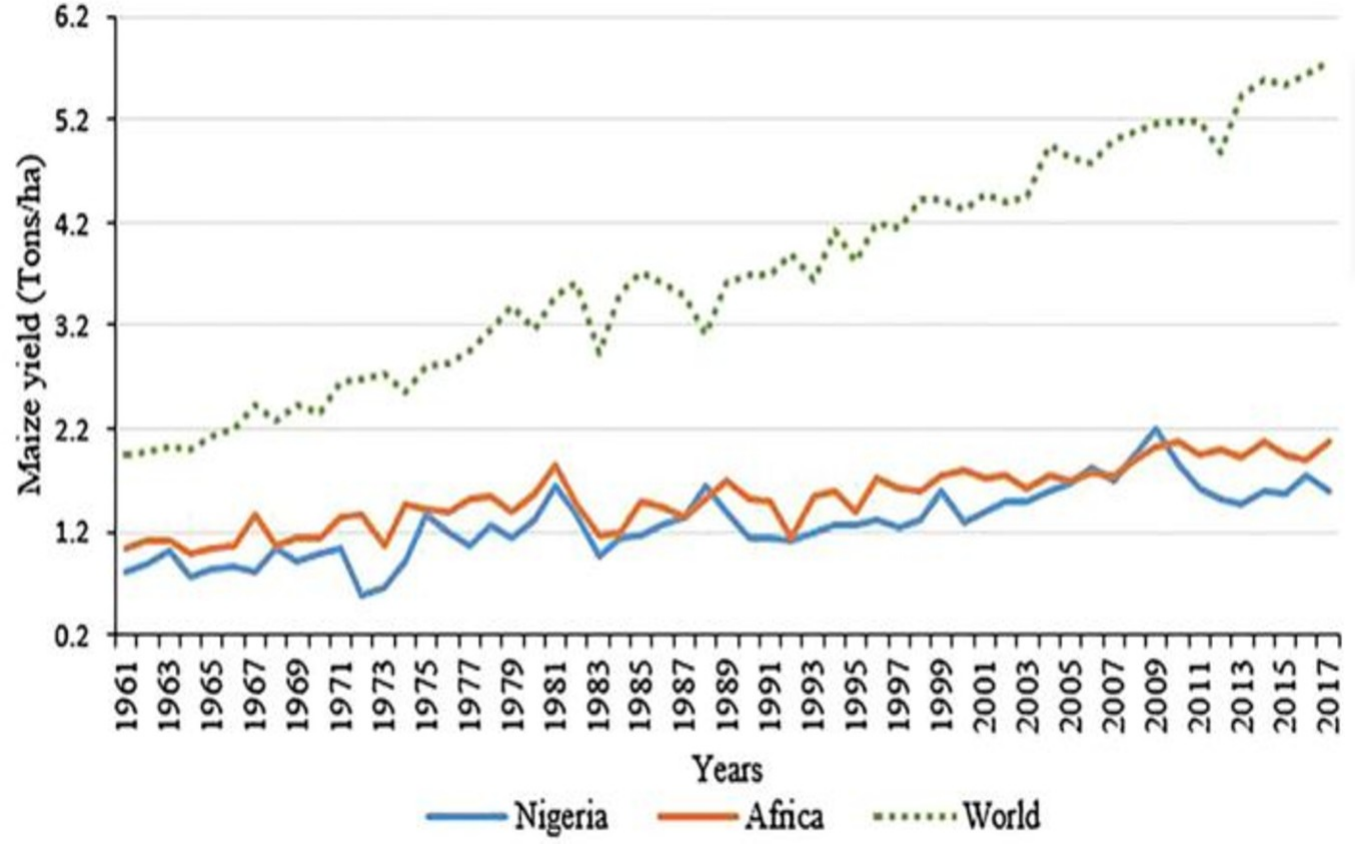
Trends of maize yield in Nigeria, Africa and the globe [1].

Despite that Nigeria is leading the continent in maize production coupled with the established importance of the crop, on-farm maize yield in the country is still less when compared to the productivity level that is attainable in standard experimental fields [29]. As reported in Fig 1, maize productivity in Nigeria and Africa as a whole has steadily lagged behind the world average, while in the last 10 years the mean yield of maize in Nigeria has been lesser than Africa’s average [1]. Given that the maize farming sector in Nigeria is mainly dominated by small scale rural farmers, the country’s performance in terms of yield may be attributed to constraints such as unavailability of modern technologies [30, 31], access to finance and extension services, and productive inputs [4, 32] which may have implications on farm incomes and food security status of rural households. To address these constraints, rural farmers have resorted to forming self-help groups, an action that dates back to 1926 in Nigeria when cocoa farmers formed farmer groups in small numbers to harness resources in selling their farm produce. In rural Nigeria, farmers also pull resources together in addressing their collective problems. For example, reduction of transaction cost through collective action is a common practice among both formal and informal farmer groups in developing countries [3].

There are existing studies that have established the importance of rural producer organisations in solving collective farmers problems such as reducing transaction costs through collective action [3], with specific emphasis on its relevance in developing countries (e.g Ito et al. [9] for China, Latynskiy and Berger [33] for Uganda and Abebaw and Haile [2] for Ethiopia). More importantly, there is empirical evidence that showed that an increase in farm revenue, as well as an improvement in the economic welfare of farmers, can be enhanced by their participation in agricultural cooperatives through increased TE and yield [10, 11, 34]. Farmers participation in both formal and informal associations can facilitate access to inputs and high yield-enhancing improved technological innovations such as pesticides, improved seed varieties, irrigation facilities, and fertilisers [4, 35, 36]. The use of improved technologies ensures that an increase in technical efficiencies of farmers and yield are achieved through an increase in the optimal combination and use of inputs [30, 37].

There are additional strands of literature that established that agricultural cooperatives influence market bargaining power and output prices which is a key motivation for farmers to increase output, productivity levels, and economic welfare. Rural farmers obtain relevant information on market prices and marketing channels through various associations to which they belong, thereby helping them with the sales of their produce and to realise higher price margin [10, 21, 34, 38, 39]. In summary, agricultural cooperatives through the provision of access to improved technologies and inputs facilitate optimal use of inputs which leads to increase in TE of farmers and consequently improved farm yield, while also through the provision of marketing information agricultural cooperatives ensures yield is better marketed thereby resulting to increased farm income and consequently overall welfare of agricultural households.

Despite established positive impacts of farmers participation in agricultural cooperatives, it is argued that there are associated costs that farmers incur when they join, including membership fees, and extracurricular activity fees among others. This is indeed the case for farmers that join farmer groups not only for strictly economic gains but for the maintenance of social order within the farming community. Besides, farmers that belong to the poorest group in the community may be excluded from fully taking advantage of cooperatives because they are unable to meet the financial commitments required for active participation. Although it is established that agricultural cooperatives impact TE and yield positively, it is potentially contingent on the associated cost of membership as well as the socio-economic characteristics of members. This study, therefore, aims at contributing to the existing literature by not only revisiting the impact of agricultural cooperatives on TE of maize farmers in rural Nigeria but also sheds more light on the scale differential impacts of membership.

## 3. Conceptual framework and estimation strategy

### 3.1. Agricultural cooperative membership decision

Farmer’s decision to participate in agricultural cooperatives or not is assumed to be a binary choice, and such a decision is constrained by many factors, for example, the availability of resources and information [6, 40]. Given that a farmer is rational in the decision-making process, and therefore will always act to maximise the potential benefits of cooperative membership. Hence, we model the likelihood of a farmer to join cooperatives as a constrained optimisation framework. Instinctively, a farmer will decide to join cooperatives if the expected benefits $\left(C_{M}^{*}\right)$ from joining exceeds the benefits from not joining $\left(C_{N}^{*}\right)$, that is, $\left(C_{i}^{*}\right)$, = $C_{M}^{*}‐C_{N}^{*}$ *> 0*. Nonetheless, $C_{i}^{*}$ variable is unobservable and only the actual participation in cooperatives can be observed. Thus, $C_{i}^{*}$ is modelled in a latent variable framework as a function of observable factors as follows

$$C_{i}^{*}=\varphi^{\prime }G_{i}+\vartheta_{i},C_{i}=1[C_{i}^{*}>0],$$ (1)

where *C*_*i*_ represents cooperative membership status which takes the value of 1 if the farmer is a member and zero if otherwise. *C*_*i*_ denotes a vector of observable farm managerial, socio-economic, and plot-specific factors that can influence cooperative membership decision. *φ* indicates a set of unknown parameters to be estimated, *ϑ* is the random error with [0, *σ*^2^] distribution. In particular, we employed a standard probit model to estimate the factors influencing farmer’s decision to participate in agricultural cooperatives. This is specified as follows

$$Pr(C_{i}=1)=Pr(C_{i}^{*}>0)=Pr(\vartheta_{i}>-\varphi G_{i})=1-H(-\varphi G_{i}),$$ (2)

where *H* denotes the cumulative distribution function for *ϑ*_*i*_. Generally speaking, the decision-making process of farmers is complex and quite heterogenous in nature, and therefore not all of the farmers will be a member of a cooperative organisation. However, it is expected that the TE of cooperative members will be higher than that of non-members [16, 17].

### 3.2. Stochastic production frontier model

The main aim of this paper is to examine the impact of agricultural cooperatives on TE of maize production. Previous studies that have estimated technical efficiencies have either used a non-parametric approach such as data envelopment analysis (DEA) [e.g., [41, 42]] or a parametric approach such as stochastic production frontier (SPF) model [e.g., [18, 43]]. However, the non-parametric DEA approach fails to account for stochastic errors and also attributes the shift from the efficiency frontier to inefficiency [44, 45]. The DEA approach is also quite sensitive to outliers and this may affect the precision of results negatively. Given that agricultural production process is affected by unpredictable climatic factors such as changes in precipitation patterns, drought, and flood [46, 47], it is expedient to adopt a parametric approach. On this basis, in line with Abdul-Rahaman and Abdulai [18] and Adebayo et al. [43], we adopt the SPF model to achieve the objective of this study. We based the SPF estimation in our study on the assumption that maize producers in rural Nigeria are exclusively either members of agricultural cooperatives or not. Generally, the SPF framework is specified as

$$Y_{i}=f(X_{i},C_{i})+\varepsilon_{i},\;given\;that\;\varepsilon_{i}=v_{i}-u_{i},$$ (3)

where *Y*_*i*_ denotes maize output of the *i*^*th*^ farmer, *X*_*i*_ is a set of productive input variables and other independent variables, *C*_*i*_ represents the binary variable that measures the impact of agricultural cooperative membership (1 = member; 0 = non-member), *ε*_*i*_ is the error term, composed of two components. The first component *v*_*i*_ is the two-sided error term and the second part *u*_*i*_ is the one-sided error term measuring efficiency. The deficiency of the production function specified in Eq (3) is that it assumes that all maize producers (i.e. both cooperative members and non-members) are not different in their access to technology. Indeed, this assumption does not hold in our case because maize farmers choose whether or not to participate in cooperatives which may be influenced by several observed and unobserved factors [4, 16, 18]. As a result of self-selection, cooperative members and non-members may face different production frontiers and, in this case, the agricultural cooperative dummy variable (*C*_*i*_) in Eq (3) is endogenous. Hence, to obtain unbiased and consistent estimates of the impact of cooperative membership on TE of maize farmers, it is necessary to model a variant of SPF that can address self-selection bias.

### 3.3. Addressing selection bias in stochastic production frontier model

Given that the decision of a maize farmer to join agricultural cooperatives or otherwise is binary in our context, there is a likelihood that some observed and unobserved factors that influence membership decision may also affect TE and yield. Simply put, there is a likelihood that the error component in the selection equation is correlated with the conventional random error in the SPF, resulting to possible self-selection bias, which has to be tackled in order to obtain consistent and unbiased parameter estimates of the causal impact of cooperative membership. To address this estimation issue, we adopt a multi-step procedure as employed in previous studies by Bravo-Ureta et al. [58], González-Flores et al. [37], Villano et al. [52], Lawin and Tamini [48], Ma et al. [16], and Abdul-Rahaman and Abdulai [18]. The first stage involves the application of the propensity score matching (PSM) to correct for the selection bias due to observable attributes. This is then followed by the application of Greene [22]’s SPF corrected for sample selection bias that may arise from unobserved attributes.

The PSM approach is used to construct a counterfactual group of farmers in such a way that it makes it possible to match the group of farmers that are cooperative members with non-members based on observable time-invariant characteristics so that both groups are as similar as possible in their features except for participation in cooperatives. In the situation where there is no baseline data for the selection of appropriate variables, Caliendo and Kopeinig [49] suggested that variables included in the PSM procedure should not vary over time and or unaffected by a change in cooperative membership. The first step in conducting PSM is to employ a binary choice model (such as logit or probit) to estimate propensity scores for all observations (in this case, for members and non-members) in the sample. The scores generated, which indicate the likelihood of being a member of agricultural cooperatives, are then employed to match members with non-members, based on a vector of observed time-invariant covariates.

Several sample selection techniques with different assumptions have been developed in past studies to address selection bias arising from unboservables within SPF models. For example Greene [22], Kumbhakar et al. [50], and Lai et al. [51]. In specific, Kumbhakar et al. [50] and Lai et al. [51] based their approaches on the assumption that selection bias stems from the correlation between the error term (*ϑ*_*i*_) in the sample selection in Eq (1) and the errors terms in the conventional SPF model, *ε*_*i*_ and *u*_*i*_, respectively while Greene [22] assumes that the selection bias is rather from the correlation between *ϑ*_*i*_ and the two-sided error term *v*_*i*_ in the conventional SPF model. The approach proposed by Greene [22] is considered to have extended the Heckman’s approach for correcting sample selection in linear models to the SPF model. Because the computation of the log-likelihood functions in Kumbhakar et al. [50] and Lai et al. [51]’s approaches is demanding, we employed the sample selection SPF model proposed in Greene (2010)’s study to correct for unobserved attributes based on the error structure presented in Eq (4). It is important to state that there is not so much superiority among the Greene [22], Kumbhakar et al. [50] and Lai et al. [51] approaches, but the assumption about the channel through which selection residual affects the frontier residual is more substantive in our choice.

$$\begin{array}{c} \mathrm{Sample}\;\mathrm{selection}:\;C_{i}=1\left[\varphi^{\prime }G_{i}+\vartheta_{i}>0\right],\;\vartheta_{i}∼N[0,\;1]\\ \mathrm{SPF}:\;y_{i}=\alpha^{\prime }X_{i}+\varepsilon_{i},\;\varepsilon_{i}∼N\left[0,\;\sigma_{\varepsilon }^{2}\right]\\ \left(y_{i},X_{i}\right)\;\mathrm{can}\;\mathrm{only}\;\mathrm{be}\;\mathrm{observed}\;\mathrm{only}\;\mathrm{when}\;C_{i}=1\\ \mathrm{Error}\;\mathrm{structure}:\;\varepsilon_{i}=v_{i}-u_{i}\\ u_{i}=\left|\sigma_{u}\;U_{i}\right|=\sigma_{u}\left|U_{i}\right|,\;\mathrm{where}\;U_{i}∼N[0,\;1]\\ v_{i}=\sigma_{v}V_{i},\;\mathrm{where}\;V_{i}∼N[0,\;1]\\ \left(\vartheta_{i},v_{i}\right)∼N_{2}\left[(0,1),\left(1,\rho \sigma_{v},\sigma_{v}^{2}\right)\right] \end{array}$$ (4)

where *C*_*i*_ represents a dummy variable with a value of one for cooperative members and zero for non-members, *G*_*i*_ denotes a set of independent variables in the sample selection equation, *ϑ*_*i*_ is the unobservable error term, *y*_*i*_ represents maize output, *X*_*i*_ is a vector of production inputs in the SPF, and *ε*_*i*_ is the composite error term. The unknown parameters to be estimated are *φ* and *α* in the selection model and SPF model, respectively. The elements contained in the error structure are items regularly included in the SPF model. The parameter *ρ* denotes the absence or presence of selectivity bias. If *ρ* is significant, it implies the presence of selection bias stemming from unobserved factors [48, 52]. The estimation of the parameters in the model is based on the traditional gradient-based Broyden-Fletcher-Goldfarb-Shanno (BFGS) technique, while the Berndt-Hall-Hall-Hausman (BHHH) algorithm estimator was used to obtain asymptotic standard errors [Greene [22] contains details regarding structure and estimation of the model].

### 3.4. Meta-frontier approach

A salient drawback of the analytical framework proposed in Greene [22] is that it is impossible to directly compare TE scores of members with non-members because the estimated TE scores are relative to each group’s frontier [37]. This is largely attributed to the fact that TE across groups that have different technologies cannot be compared directly. An attempt to tackle this deficiency involves employing the meta-frontier production function approach for the construction of a common benchmark for a valid comparison between members and non-members.

Using the matched samples, we compute a meta-frontier that envelops the deterministic component [52] of the cooperative members and non-members for the sample selection models. This approach allows for the estimation of the gaps between the meta-frontier and the individual group frontiers, referred to as the meta-technology ratio (MTR).

The meta-frontier is specified as follows

$${y_{i}}^{*}=f(X_{i},\alpha^{*})=e^{X_{i}\alpha^{*}}$$ (5)

where *y*_i_* represents the meta-frontier output, α* indicates the vector of parameters to be estimated given that *X*_*i*_a*≥*X*_*i*_*α*_*j*_ and *α*_*j*_ are parameters obtained from the member and non-member group frontiers. According to Villano et al. [52], the MTR can be expressed as the ratio of the highest attainable group output to the highest possible meta-frontier output. The MTR lies between zero and one, and is expressed as

$$MTR=\frac{e^{X_{i}\alpha_{j}}}{e^{X_{i}\alpha^{*}}}$$ (6)

The TE with respect to the meta-frontier (MTE) is therefore computed as

$$MTE={TE}_{j}*{MTR}_{j}$$ (7)

To compute the meta-frontier, we employed the parametric stochastic frontier procedure developed by Huang et al. [53] which involves the application of the quasi–maximum likelihood estimation framework. The advantages of this approach over the linear programming (deterministic) approach proposed by O’Donnell et al. [54] are that Huang et al. [53]’s approach produces statistical inferential estimates while it also allows the random shocks to be separated from the technology gaps [48]. In particular, the predicted values of the results obtained from the estimates of the group-specific frontier are pooled to compute the stochastic meta-frontier. We conducted the analyses using STATA statistical software.

### 3.5. Empirical model specification

As explained in the previous section, we employed a multi-step approach that involves the application of both the PSM and selectivity-corrected SPF estimation techniques to address self-selection biases from observed and unobserved attributes, respectively. The first step involves the implementation of PSM which requires a matching procedure. The use of PSM does not completely remove selection bias that arises from observed characteristics across the group of maize farmers that are members and non-members, however, Imbens and Wooldridge [55] argued that PSM produces results that are fairly reasonable and accurate. Many matching procedures can be employed to match members and non-members based on similar observed time-invariant characteristics, including radius matching, stratification matching, nearest neighbour matching, and kernel-based matching [see Cameron and Trivedi [56] and Caliendo and Kopeinig [49] for a detailed explanation of these matching procedures]. In our study, we choose to adopt the “1-to-1 nearest neighbour without replacement” matching algorithm in which every maize farmer that is cooperative member is matched with non-member by imposing the common support condition [57]. The justification for employing the “1-to-1 nearest neighbour without replacement” criterion is premised on its ease of implementation [49, 58]. Also, compared with other matching criteria, it can be easily interpreted intuitively [22]. The application of this matching procedure has become a widely accepted choice in applied economics literature [16, 58]. It is important to emphasize that we also applied other matching procedure particularly an Epanechnikov kernel matching, and based on a balancing test, the nearest neighbour produced the best-matched samples.

The next step after conducting the matching procedure is to estimate a corrected sample selection bias SPF model. To implement this model, it is required to estimate decision of *i*^*th*^ farmer to join cooperatives or otherwise (because the model will only include matched samples, it may be referred to as probit sample selection model [52]), a behaviour which can be explained by a criteria function, and expressed as a function of exogenous farm and farmers socioeconomic factors (*G*) as follows

$$C_{i}=\varphi_{o}+\sum_{j=1}^{22}\varphi_{j}G_{ij}+\vartheta_{i},$$ (8)

where *C*_*i*_ is a dummy variable equal 1 for cooperative members, and zero if otherwise, *G* is a set of exogenous variables influencing farmer’s decision to join cooperatives, and *ϑ*_*i*_ indicates the unobservable statistical noise term which follows *N*(0, *σ*^2^) distribution. The set of exogenous variables in *G* are gender, age, age squared, household size, education, land ownership, asset value, access to irrigation, farm size, risk preference, access to extension, access to credit, distance to seed market, row planting practice, soil and water conservation practice, intercropping practice, soil fertility, drought experience, and regional factors.

There is an endogeneity issue that needs to be addressed in Eq (8) to obtain consistent and unbiased estimates. Maize farmers access to credit facilities can be potentially facilitated through cooperative membership. This is the case in Nigeria as farmers are usually required to be a member of at least one farmers group to secure loans or finance needed for farming activities as well as for expansion. In the same vein, access to extension services may be enhanced for members of cooperative societies. For these reasons, it is necessary to treat access to extension services and credit variables as potentially endogenous in the model [14, 18]. Following the approach suggested by Wooldridge (2015), we address this endogeneity issue by estimating a two-stage control function model. In the first stage, we modelled access to credit and extension services as a function of appropriate instruments and other explanatory variables in the cooperative membership probit model [18]. The selected instruments must be valid, that is, they should be strongly correlated with access to credit and extension services but not with maize farmer participation decision. We employed television/radio coverage for information (access = 1, 0 if otherwise) and awareness of credit sources information for improved maize varieties (aware = 1, 0 if otherwise) as instrumental variables for access to extension services and credit, respectively (See S1 Table in the S1 Appendix). It is expected that farming households that are not aware of sources of credit such as local microfinance banks or perhaps live farther away from centres for credit sources are less likely to access credit, while access to extension services could be well facilitated by television/radio coverage for information sharing. However, the two instrumental variables are arguably outside the household’s cooperative membership decision. The second stage involves incorporating the observed values of access to credit and extension variables as well as their corresponding residuals predicted from the first-stage into the cooperative membership decision equation.

There are several functional forms employed in agricultural production economics studies for analysing efficiency, however, the duo of Cobb-Douglas (CD) and translog (TL) are mostly employed [58, 59]. We conducted a maximum likelihood ratio (LR) test to compare these two functional forms. The LR test statistic was estimated using the formula: *LR* = −2{*ln*[*H*_0_]−*ln*[*H*_1_]}, where *H*_0_ was assumed by the value of log-likelihood for CD and *H*_1_ represented the alternative hypothesis and was assumed by the value of log-likelihood for the TL model. Based on the results of the LR test (*LR* = 31.25, *p-value* = 0.214), the alternative hypothesis (*H*_1_) was rejected in favour of CD functional form at 5% level of significance. While the CD model is said to be nested in the TL model and restrictive, it produces estimates that are more satisfactory [18]. Besides, there are often multicollinearity issues that arise from input variables and their interaction terms in TL functional form [60]. Therefore, we adopted the CD functional form for our SPF analysis. This is specified as

$$ln(Y_{i})=\beta_{o}+\sum_{k=1}^{5}\beta_{j}{lnX}_{ji}+\sum_{k=1}^{8}\delta_{k}D_{ki}+v_{i}-u_{i},\;only\;if\;G_{i}=1$$ (9)

where *ln* denotes natural logarithm, *Y*_*i*_ is output of *i*^*th*^ maize farmer, *X*_*ji*_ represents a set of inputs; *D* is the binary variables; *β*_*j*_ and *δ*_*k*_ are the unknown parameters to be estimated; *v*_*i*_ and *u*_*i*_ indicate the two components of the composed random error. Following Bravo-Ureta et al. [58] and Ma et al. [16], the dependent variable in the SPF model is the value of maize output measured in Nigerian Naira ($1 = NGN 284 at the time of the survey). Unlike previous studies [e.g. Abdul-Rahaman and Abdulai [18] and Adebayo et al. [43]] that employed yield as a dependent variable, the use of value of maize output allows adjustment for inherent quality variations in maize output such as grain weight, size and colour and solid content [61]. This is justifiable in our study because farmers cultivate more than 36 varieties of maize. The independent variables include five traditional production inputs and eight binary variables. The production inputs are expenditures on purchased inputs (seeds, fertiliser and chemicals); value of hired labour; and cultivated area. The binary variables are fertiliser, chemical, soil quality, and regional dummies. One issue with input variables is that not all farmers use some inputs, for example, many farmers may not use hired labour for maize production, therefore the logarithm transformation of this input variable will yield many missing values. To address the zero values of inputs, we follow Battese [62]’s procedure by including dummies for input variables, in such a way that the log-transformation of inputs with zero values is executed only if it is positive, and zero otherwise [16, 18, 52].

## 4. Data and descriptive statistics

In this study, we employ a cross-sectional dataset collected from farm household survey conducted between November 2014 and February 2015 in Nigeria under the Drought Tolerant Maize for Africa (DTMA) project coordinated by the International Institute of Tropical Agriculture (IITA). The survey was designed to evaluate the impact of adoption of improved maize varieties on productivity and farmers' wellbeing. The sample was drawn through a multistage stratified random sampling procedure across all the 36 states in Nigeria in order to obtain a nationally representative sample of maize producing farming households. The first stage involves dividing the 36 states into five homogenous sub-groups based on the total land area dedicated to maize production per state. Out of the five sub-groups, 18 states were selected randomly. The second stage involves a random selection of enumeration areas (EAs) in each of the selected states. In this stage, the National Population Commission (NPC) provided the sampling frame of all the EAs in each of the states. The totals of EAs obtained from the NPC were thereafter divided by the Local Government Areas (LGAs) into each of the selected states to obtain EAs per LGAs. The agricultural development programs (ADPs) office in Nigeria provided the list of all farmers producing maize for the selected EAs per LGA. Lastly from the list of all the maize farming households, five farmers were selected randomly for interviews per each of the EA. In total, the number of farming households that form the sampling observations is 2,228 comprising 1,416 farmers with at least one agricultural cooperative membership and 811 farmers without any cooperative membership. A broad range of information is contained in the survey including farming household’s socio-economic characteristics, expenditure on food and non-food items, income from maize enterprise, as well as input and output variables (such as value of maize output, land area cultivated, labour units). Table 1 presents the description and summary statistics of these variables. Maize farmers that are members of agricultural cooperatives are about 64% of the pooled samples. On average, farmers in the pooled samples are still in their economic active age of about 48 years old, who may have had at least 7 years of formal education, farm on 4.4ha, and produce about 3879.2Kg of maize.

**Table 1:** Description and summary statistics of variables of the pooled samples.

Variables	Notation	Description	Mean (Std. Dev.)
**Probit Model**			
*Farm managerial and socioeconomic factors*			
Gender	*G*_1_	if the farmer is male, 0 otherwise	0.89 (0.31)
Age	*G*_2_	Age of the household head in years	47.95 (13.24)
Household size	*G*_3_	Number of family members	6.95 (2.97)
Education	*G*_4_	Number of years of formal education by the farmer	7.45 (5.78)
Owned land	*G*_5_	1 if the farmer owned land, 0 otherwise	0.86 (0.35)
Log of asset value	*G*_6_	Value of asset in logarithm form	12.50 (1.71)
Irrigation	*G*_7_	1 if the farmer has access to irrigation, 0 otherwise	0.12 (0.33)
Risk	*G*_8_	1 if the farmer is willing to try new things, 0 otherwise	0.73 (0.44)
Access to extension	*G*_9_	1 if the farmer has access to extension, 0 otherwise	0.11(0.31)
Access to credit	*G*_10_	1 if the farmer has access to credit, 0 otherwise	0.16 (0.36)
Distance to seed market	*G*_11_	Distance of farmer to seed market in kilometres	17.59 (10.04)
*Plot-specific factors*			
Row planting	*G*_12_	1 if the farmer practices row planting, 0 otherwise	0.81 (0.38)
Water conservation	*G*_13_	1 if the farmer practices water conservation, 0 otherwise	0.54 (0.49)
Intercropping	*G*_14_	1 if the farmer practices intercropping, 0 otherwise	0.51 (0.49)
Good soil	*G*_15_	1 if the farmer farms on good soil, 0 otherwise	0.73 (0.44)
Drought	*G*_16_	1 if the farmer’s field is prone to drought, 0 otherwise	0.18 (0.38)
*Regional dummies*			
North east zone	*G*_17_	1 if farm is located in North east zone, 0 otherwise	0.04 (0.21)
North west zone	*G*_18_	1 if farm is located in North west zone, 0 otherwise	0.35 (0.47)
North central zone	*G*_19_	1 if farm is located in North central zone, 0 otherwise	0.27 (0.44)
South east zone	*G*_20_	1 if farm is located in South east zone, 0 otherwise	0.04 (0.19)
South south zone	*G*_21_	1 if farm is located in South south zone, 0 otherwise	0.05 (0.21)
South west zone	*G*_22_	1 if farm is located in South west zone, 0 otherwise	0.24 (0.42)
**SFP Model**			
Output	*Y*	Value of total production of maize in Nigerian Naira (NGN)	253875.00 (329518.10)
Area	*X*_1_	Total maize area planted in hectares	4.42 (3.19)
Seed	*X*_2_	Value of seed used in NGN	1241.66 (2946.93)
Fertiliser	*X*_3_	Value of fertiliser used in NGN	30040.35 (35725.27)
Chemical	*X*_4_	Value of chemical used in NGN	12242.36 (23929.14)
Labour	*X*_5_	Value of hired labour used in NGN	58971.29 (81485.16)

Note: $1 is equivalent to NGN 284 at the time of the survey.

**Fig 2:**
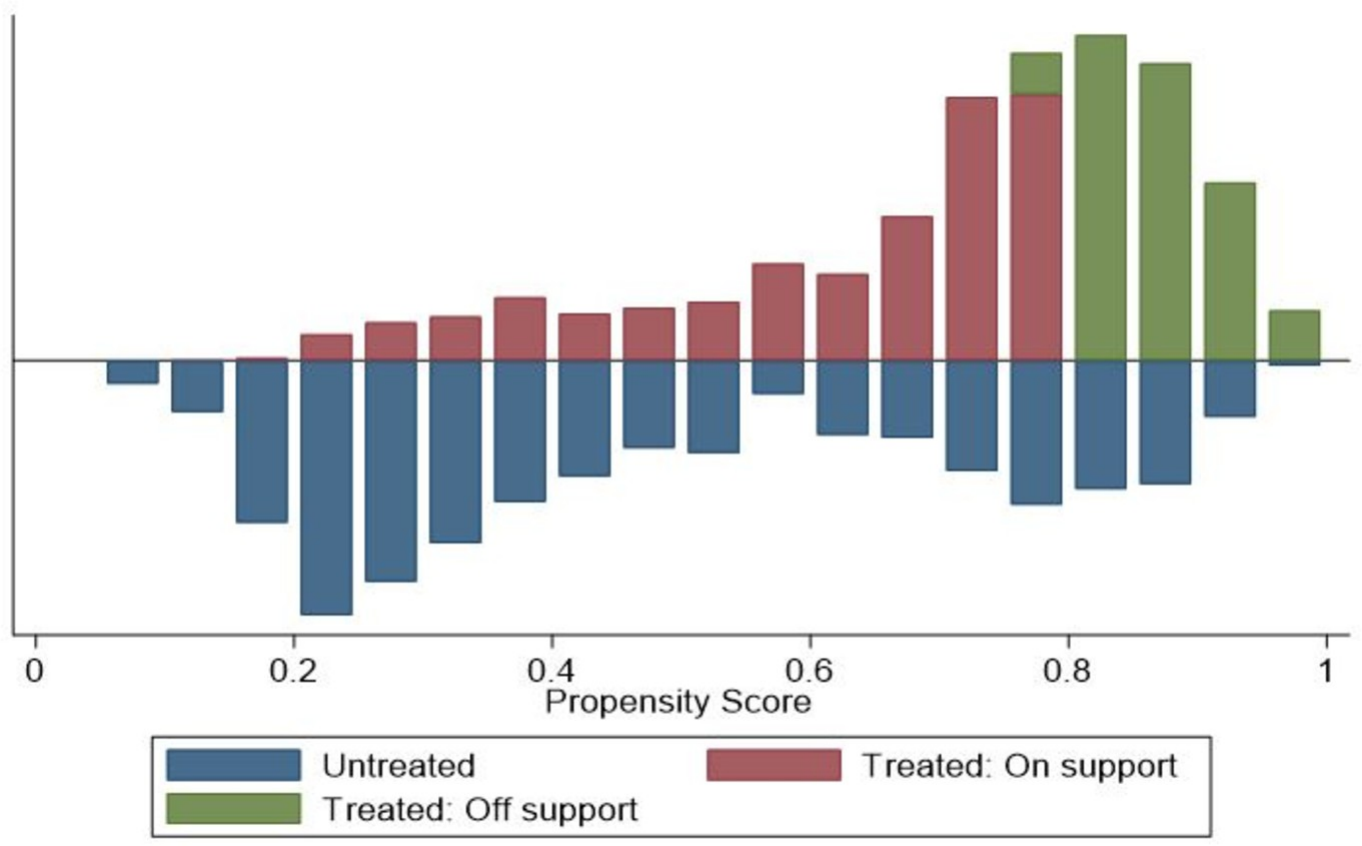
Density of the propensity scores and common support graph for cooperative members and non- members.

As stated in the previous section, the PSM analysis requires a matching procedure using time-invariant observable attributes. The “1-to-1 nearest neighbour without replacement” matching procedure produced a total of 1,622 matched observations, 811 for both members and non-members (see Fig 2 for the common support graph). Table 2 presents the summary statistics of all variables in the matched and unmatched samples. The mean differences between members and non-members, as well as the statistical *t*-tests for matched and unmatched samples, are also reported in Table 2. The comparisons of means for the variables of the matched samples reveal that the means of observable characteristics of members are not significantly different from non-members, suggesting that the balancing condition of the selected control variables is fulfilled [57]. Conversely, for the majority of the variables for the unmatched observations, we observe significant mean differences between members and non-members. The results show that cooperative members are older, more educated, have higher asset value, higher access to extension and credit facilities and are more willing to try new things, perceived to farm on fertile soil, and live farther away from seed markets, compared to farmers that are non-members. Cooperative members also tend to practice row planting, intercropping, and water conservation more, as well as produce higher maize yield while they also use more inputs including land area, labour, seed, fertiliser, and chemicals. The proportion of farming households with access to extension and credit tends to be significantly higher for members than non-members.

**Table 2:** Summary statistics of variables used in the probit and stochastic frontier model using unmatched and matched samples.

Variables	Unmatched samples			Matched samples		
	Cooperative members	Cooperative non-members	Mean diff.	Cooperative members	Cooperative non-members	Mean diff.
Gender	0.89(0.31)	0.91(0.29)	-0.02	0.90 (0.30)	0.91 (0.29)	-0.01
Age	48.09 (13.04)	47.72 (13.59)	0.36	48.37 (13.94)	47.72 (13.59)	0.66
Household size	6.95 (2.98)	6.95 (2.90)	0.00	6.97 (3.16)	6.95 (2.89)	0.02
Education	8.01 (5.75)	6.48 (5.70)	1.53***	7.45 (6.00)	6.47 (5.70)	0.97
Owned land	0.84 (0.35)	0.89 (0.31)	-0.05***	0.83 (0.37)	0.88 (0.31)	-0.05
Log of asset value	12.64 (1.63)	12.25 (1.80)	0.39***	12.43 (1.64)	12.25 (1.80)	0.18
Irrigation	0.10 (0.29)	0.16 (0.37)	-0.07***	0.11 (0.32)	0.17 (0.37)	-0.06
Risk	0.76 (0.43)	0.67 (0.47)	0.09***	0.71 (0.45)	0.67 (0.47)	0.04
Access to extension	0.13 (0.33)	0.06 (0.25)	0.07***	0.07 (0.26)	0.06 (0.24)	0.01
Access to credit	0.20 (0.40)	0.08 (0.28)	0.12***	0.08 (0.28)	0.08 (0.27)	0.00
Distance to seed market	18.33 (10.90)	16.31 (8.18)	2.02***	17.20 (10.11)	16.31 (8.18)	0.89
Row planting	0.82 (0.38)	0.78 (0.41)	0.04**	0.77 (0.42)	0.78 (0.41)	-0.01
Water conservation	0.55 (0.49)	0.51 (0.50)	0.04**	0.49 (0.50)	0.51 (0.50)	-0.02
Inter cropping	0.54 (0.49)	0.46 (0.49)	0.08***	0.52 (0.49)	0.46 (0.49)	0.06
Good soil	0.72 (0.45)	0.74 (0.43)	-0.02	0.68 (0.46)	0.74 (0.44)	-0.05
Drought	0.20 (0.40)	0.15 (0.36)	0.05***	0.18 (0.38)	0.15 (0.36)	0.03
Output	265378.90 (331896.90)	247290.90 (328084.80)	18088.05	269582.50 (353125.00)	265378.9 (331896.90)	4203.59
Area	4.25 (3.10)	4.70 (3.33)	-0.45***	4.32 (3.13)	4.70 (3.33)	-0.38
Seed	1219.46 (2964.68)	1280.44 (2917.06)	-60.97	7374.58 (1534.30)	7300.67 (1093.02)	73.91
Fertilizer	26629.94 (35157.24)	35999.1 (35949.42)	- 9369.16***	30423.87 (39143.92)	36302.80 (35648.77)	-5878.93
Chemical	13471.99 (25147.21)	10093.92 (21483.54)	3378.07**	14925.70(17094.95)	14368.72 (20724.56)	556.98
Labour	64052.49 (86334.24)	50093.27 (71423.75)	13959.22***	55995.39 (78212.76)	50357.14 (71239.58)	5638.24
North east zone	0.06 (0.24)	0.02 (0.15)	0.04***	0.03 (0.19)	0.02 (0.15)	0.01
North west zone	0.19 (0.39)	0.62 (0.48)	-0.42***	0.34 (0.47)	0.62 (0.48)	-0.28
North central zone	0.35 (0.47)	0.13 (0.33)	0.22**	0.19 (0.39)	0.13 (0.33)	0.06
South east zone	0.04 (0.20)	0.03 (0.17)	0.01***	0.05 (0.22)	0.03 (0.17)	0.02
South south zone	0.05 (0.21)	0.05 (0.21)	0.00	0.08 (0.27)	0.05 (0.21)	0.03
South west zone	0.29 (0.45)	0.15(0.35)	0.14***	0.31 (0.46)	0.15 (0.36)	0.15
Number of observations	1,417	811		811	811	

Note: Standard deviation values are in parentheses.

## 5. Results and discussion

### 5.1. Determinants of cooperative membership decision

Several studies have assessed factors influencing farmers' decisions to participate in agricultural cooperatives [e.g. [4, 10, 16, 18]]. With particular reference to maize farmers in rural Nigeria, we estimate the factors influencing cooperative membership decision, and the results for matched samples are reported in Table 3. The average marginal effects are also estimated and reported to ensure the results are better interpreted [63]. The measures of goodness of fit for the model, including the Wald *chi*^2^, Pseudo *R*^*2*^ and Archer and Lemeshow [64] are also reported. According to all the diagnostics measures, we are confident to infer that the model is a good fit.

**Table 3:** Estimates of the probit equation using matched samples.

Variables	Coefficients	Robust Std. Error	Marginal effects	Robust Std. Error
Gender	0.012	0.113	0.005	0.045
Age	0.027	0.019	0.011	0.008
Age squared	-0.000	0.000	0.000	0.000
Household size	0.099***	0.025	0.040***	0.010
Education	0.028***	0.008	0.011***	0.003
Owned land	-0.084	0.108	-0.033	0.043
Log of asset value	0.078***	0.027	0.031***	0.011
Irrigation	0.006	0.115	0.003	0.046
Farm size	-0.021	0.016	-0.008	0.006
Risk	-0.161*	0.083	-0.064*	0.033
Access to extension	0.376*	0.194	0.147**	0.073
Access to credit	0.011	0.124	0.004	0.050
Distance to seed market	0.004	0.004	0.001	0.002
Row planting	-0.025	0.086	-0.010	0.034
Soil and water conservation	-0.022	0.076	-0.009	0.030
Inter cropping	0.318***	0.083	0.126***	0.033
Good soil	-0.125	0.093	-0.050	0.037
Drought	0.529***	0.147	0.206***	0.054
Northcentral	0.260	0.392	0.103	0.153
Northwest	-1.389***	0.189	-0.512***	0.059
Northeast	0.181	0.250	0.072	0.098
South south	-0.539*	0.275	-0.206**	0.097
South east	-0.757**	0.312	-0.277***	0.096
Extension residual	0.555	0.818	0.817	1.124
Credit residual	0.740	0.959	0.492	0.782
Constant	0.292			
	(0.737)			
Wald *chi*^2^ [30]	316.241***			
Pseudo *R*^*2*^	0.31			
Goodness of fit measure Archer and Lemeshow [64]	0.416			
Observations	1,622		1,622	

Notes: Robust standard errors in parentheses

*** *p*<0.01

** *p*<0.05

* *p*<0.1.

We omitted the results related to unmatched samples but can be made available from authors upon request.

The results show that the probability of being a cooperative member increased significantly with the size of households. Rural farm households with large size do have a ready supply of labour for planting and other farming practices that could facilitate expansion, thereby necessitating farmers to join cooperatives for easy access to inputs such as seeds, fertilisers, and chemicals [46]. *Ceteris peri bus*, households with higher levels of education have 1.1% probability of participating in agricultural cooperatives. Education facilitates the acquisition of new information such as market and input prices, which appears to support the reason why farmers may likely participate in cooperatives [16, 65]. The marginal effect of the asset value variable was found to be positive and significant, implying that a unit increase in asset value will result in 3.1% likelihood of farmers to participate, consistent with Mojo et al. [65] who found that farmers that are better-off in term of asset value are more likely to participate in cooperatives.

Another important factor that influences farmers' decisions to join cooperatives is their willingness to take risk. Farming households that are willing to take the risk of trying new seed varieties are more likely to participate in cooperatives. This is expected given that many smallholder farmers join cooperatives with the expectation that membership provides seed inputs [18]. Maize farming households that have sufficient access to extension services, all things being equal, have higher likelihood to join cooperative, in line with Ma et al. [16] and Abdul-Rahaman and Abdulai [18]. Although not significant, access to credit variable was found to be positive in influencing farmers' decisions to join cooperatives, implying that farmers with lesser credit constraints are more likely to participate. These farmers are more likely to fulfil membership commitments such as periodic cash contributions and membership fees as well as meet with other production commitments such as the purchase of fertiliser and chemicals [18].

Additionally, the variable representing the adoption of intercropping was found to be positive and significantly influence farmers' decisions to join cooperatives. This suggests farmers who adopted intercropping production system are more likely to join cooperatives. The objective of intercropping is to enhance the production of more crops on a given farmland, suggesting that such farming households would likely want to join cooperatives as a strategy to cope with potential production shocks. The results also show that farmers that have experienced drought shocks in the past are more likely to participate in cooperatives. This is because these farmers are likely to have better understanding of not only the environmental challenges but also production and marketing challenges in maize farming, thereby have a higher likelihood to participate in cooperatives. All the dummy variables representing the six regions in Nigeria (with Southwest region as reference) significantly influence farmers’ decisions to join cooperatives, but with different signs. Farming households located in Northern regions are more likely to join while farmers located in southern regions are less likely to participate. Ma et al. [16] and Abdul-Rahaman and Abdulai [18] also established that location of farmers plays an important role in farmers participation decisions. Finally, the coefficients of the generalized residuals predicted from the first stage of the control function for access to credit and extension are also reported in Table 3. The coefficients of the two residuals are not statistically significant, suggesting that both access to credit and extension are not endogenous in the cooperative membership decision model.

### 5.2. Results of the production functions

In Table 4, we report the maximum likelihood estimates of the conventional and selectivity- corrected SPF using the matched samples. The estimation is based on a CD production SPF, in which the output and input variables are in natural logarithmic forms. Hence, the coefficients of input variables can be interpreted as partial elasticities [66]. To investigate if there are technology differences between members and non-members, we conducted a likelihood ratio (LR) test. The LR test is estimated as follows: LR = −2{lnL_p_−(lnL_m_+lnL_nm_)}, where lnL_p_, lnL_m_ and lnL_nm_ indicate the values of log-likelihood obtained from the pooled samples, two separate SPFs models for members and non-members, respectively. The test is based on the null hypothesis that there is no difference between the pooled frontier model and the two group frontiers [58]. Based on the results of generalised likelihood ratio test statistic (*LR* = 75.42, *p-value* = 0.000), the null hypothesis of homogenous technology between members and non-members is rejected at 1% level of significance; therefore, necessitating that separate frontiers for members and non-members should be estimated.

**Table 4:** Maximum likelihood estimates of the conventional and sample selection SPF models using matched samples.

Variables	Conventional SPF						Selectivity-corrected SPF			
	Pooled		Members		Non-members		Members		Non-members	
	Coeff.	S.E	Coeff.	S.E	Coeff.	S.E	Coeff.	S.E	Coeff.	S.E
ln seed	0.268	0.225	0.378	0.296	0.146	0.351	0.379	0.296	0.156	0.352
ln maize land	0.163***	0.039	0.209***	0.055	0.138**	0.054	0.209***	0.055	0.137**	0.054
ln hired labour	0.111***	0.015	0.097***	0.022	0.121***	0.020	0.098***	0.022	0.123***	0.021
ln fertiliser	0.078***	0.022	0.099***	0.032	0.055*	0.032	0.098***	0.033	0.051	0.034
ln chemical	0.130***	0.030	0.119***	0.043	0.138***	0.042	0.119***	0.043	0.138***	0.042
Fertiliser dummy	-0.031	0.079	-0.146	0.114	0.057	0.111	-0.147	0.115	0.047	0.113
Chemical dummy	0.171**	0.067	0.274***	0.098	0.083	0.092	0.275***	0.098	0.082	0.092
Soil quality dummy	0.068***	0.023	0.171**	0.085	0.047*	0.024	0.169*	0.087	0.050*	0.026
Irrigation dummy	0.005	0.079	0.001	0.124	0.043	0.101	0.017*	0.009	0.037*	0.020
Cooperative membership	0.102***	0.034	-	-	-	-	-	-	-	-
Constant	6.830***	2.013	5.506**	2.731	8.225***	3.153	5.495**	2.728		
Lambda (λ)	0.795***	0.295	0.425***	0.084	1.111***	0.257	-	-	-	-
Sigma-u (*σ*_*u*_)	-	-	-	-	-	-	0.462	0.101	0.959***	0.187
Sigma-v (*σ*_*v*_)	-	-	-	-	-	-	1.066***	0.125	0.858***	0.074
Selectivity correction term [*ρ*_(*w*,*v*)_]	-	-	-	-	-	-	0.024**	0.012	0.065*	0.036
Regional fixed effects	YES		YES		YES		YES		YES	
Log-likelihood function	-2413.56		-1229.49		-1176.80		-1229.49		-1176.70	
Observations	2,228		1,417		811		811		811	

Notes

*** *p*<0.01

** *p*<0.05

* *p*<0.1.

We omitted the results related to unmatched samples but can be made available from authors upon request.

Estimated results presented in Table 4 show that the partial elasticities of all the inputs variables are positive, albeit differ in magnitudes and levels of statistical significance. With a particular reference to the pooled estimation, the results show that cooperative membership is positive and significant, suggesting that membership is positively related to higher maize output. Studies such as [16, 18, 65] reported similar findings. The results of all the estimated models show that land and chemical inputs contributed the highest to maize output for both cooperative members and non-members. The contribution of these inputs is positive and significant at a minimum of 5% level. The findings are consistent with previous studies conducted by [17, 18]. Although to a lesser extent relative to land and chemical inputs, hired labour input contributes positively and significantly to maize revenue for both members and non-members. Maize production by smallholder farmers in developing countries is still labour intensive, and therefore attempt to increase maize output and revenue requires reasonable investment in labour input needed for optimal production process such as the application of productivity-improving technologies [16, 24]. It is also revealed that fertiliser input contributes significantly to maize output of members but had no significant impact on non-members. Seed input contributed the least and insignificant to maize output for both members and non-members. This is likely to be attributed to the fact that adoption rates of improved maize varieties among smallholder farmers in rural Nigeria are still low [28, 46]. The dummy variables representing soil quality and irrigation both have a positive and significant impacts on the value of maize output, suggesting the relevance of soil nutrients and irrigation technology in enhancing the value of maize output. This in line with the findings that adoption of irrigation technology and soil improvement practices improves productivity [67, 68].

The estimates of selectivity-corrected SPF models in Table 4 show that the selection correction term, *ρ*_(*w*,*v*)_, is significantly different from zero for both members and non-members indicating the presence of selection bias, which may likely be attributed to factors that are unobservable but affect the cooperative membership decision, for example, farmers' managerial and production skills [16, 18]. This further justifies the adoption of SPF framework that corrects for sample selection bias, and that failure to correct for such bias will yield inconsistent TE scores which can be unreliable for policy recommendation [52, 58]. Besides, the parameter (λ) was found to be statistically and significantly different from zero at 1% level, suggesting that technical inefficiency contributes significantly to observed maize output variability. Table 5 reports the estimation of the stochastic meta-frontier for matched samples, upon which the meta-frontier gap and meta-frontier TE are estimated.

**Table 5:** Estimates of the stochastic meta-frontier using matched samples.

Variables	Coefficient	Standard Error
ln seed	0.193	0.256
ln maize land	0.177***	0.009
ln hired labour	0.102***	0.004
ln fertiliser	0.094***	0.005
ln chemical	0.127***	0.007
Fertiliser dummy	-0.030	0.019
Chemical dummy	0.176***	0.016
Soil quality dummy	0.092***	0.015
Irrigation dummy	0.043**	0.019
Constant	7.321***	0.508
Regional fixed effects	YES	
Log-likelihood function	-1,021.251***	
Observations	1,622	

Notes

*** *p*<0.01

** *p*<0.05

* *p*<0.1.

We omitted the results related to unmatched samples but can be made available from authors upon request.

### 5.3. Yield and technical efficiency scores

In Table 6, we present the results of the mean TE scores obtained from the conventional and sample selection SPF models for the pooled samples, members, and non-members for matched samples. Furthermore, the mean TE differences between members and non-members and their corresponding percentage differentials based on statistical *t*-test are also reported in Table 6. With regards to the pooled estimates (TE-Pool), the results reveal that there is no significant difference between the mean TE scores of members and non-members. As established in the early part of this paper, the null hypothesis that both members and non-members operate on the same frontier was rejected, suggesting that comparison should not be made between the two groups using the production frontier estimated from the pooled data.

**Table 6:** TE levels and differentials across the SPF models.

Item	Member		Non-member		Test of means^a^
	Mean	S.D	Mean	S.D	
TE-Pool^þ^	0.65	0.09	0.64	0.07	0.7140^*NS*^ (1.56%)
TE-Conventional SPF^þþ^	0.72	0.04	0.58	0.14	78.86*** (24.14%)
TE-Sample Selection SPF^þþþ^	0.75	0.03	0.62	0.14	73.55*** (20.96%)
Meta-frontier technology gap (MTR)	0.80	0.07	0.71	0.10	11.21*** (12.67%)
TE-Meta-frontier (MTE)^¥^	0.60	0.10	0.41	0.12	70.55*** (46.34%)

Notes

*** indicates *p*<0.01.

*NS* means not significant.

^a^ *t* test was conducted to determine if the mean TE of members is statistically different from non-members. Values in parentheses represent percentage increase used to determine the TE differential between members and non-members.

^þ^ TE estimates using the conventional SPF and the pooled dataset.

^þþ^ TE estimates relative to the individual group’s frontier using the conventional SPF.

^þþþ^ TE estimates relative to the individual group’s frontier using the Sample Selection SPF.

^¥^TE estimates relative to the meta-frontier.

Results regarding the conventional SPF estimates (TE-Conventional SPF) reveal that cooperative members operate at a mean TE level of 0.72, while non-members operate at a mean TE of 0.58, relative to their group frontiers. In the same vein, the sample selection SPF estimates (TE-Sample Selection SPF) show that the mean TE score for members is 0.75, and 0.62 for non-members, relative to their group frontiers. These findings established that cooperative members perform better by operating closer to their group frontier than non-members, suggesting that cooperative members tend to use their resources more efficiently than non-members with respect to their respective technologies.

While the estimates presented so far are important in addressing selectivity bias, the comparisons across the two groups are still not proper, and therefore to make a valid comparison between the two groups, we conducted a stochastic meta-frontier analysis by employing the approach developed by Huang et al. [53]. By so doing, we obtain the technology gaps between the meta-frontier and the individual group frontiers (usually called the meta-technology gap ratio) and the TE with respect to the meta-frontier (MTE). The estimated average meta-technology gap ratio for cooperative members (0.80) was significantly higher than non-members (0.71). Consequently, the average TE relative to the meta-frontier for cooperative members (0.60) was also significantly higher than that of non-members (0.41).

Generally, the results reflect that the TE scores are overestimated provided selectivity bias is not appropriately dealt with, suggesting that accounting for selectivity bias from both observed and unobserved attributes is important in accurately estimating the impact of cooperative membership. This evidence of overestimation bias is in line with the results obtained in Bravo-Ureta et al. [58] but in contrast with Villano et al. [52] where evidence of selection bias was the opposite.

In Table 7, we report the differences in the predicted value of output between members and non-members before and after correcting for biases due to observed and unobserved factors. This was carried out by predicting the average frontier output value obtained from the unmatched conventional SPF (without bias correction) and the matched sample selection SPF (with bias correction) models. The results show that farmers with cooperative membership obtained higher maize output value than non-members, with a percentage difference of about 45.90% when selection bias was not corrected for. But when selection bias due to both observed and unobserved variables are addressed, the percentage difference in maize output value between members and non-members stands at 55.86%, indicating that cooperative membership contributes significantly to increasing maize output value.

**Table 7:** Predicted frontier output before and after bias correction.

SPF Model	Members	Non-members	% increase in predicted output ^c^	Test of means ^d^
^*a*^ *Conventional*				
Mean	332,679.00	228,018.50	45.90%	6.190***
Minimum	85,495.90	52,449.91		
Maximum	1,060,289.00	682,770.60		
^*b*^ *Sample selection*				
Mean	364,795.40	234,049.70	55.86%	7.757***
Minimum	116,657.60	46,234.25		
Maximum	1,134,587.00	660,754.30		

Notes

*** *p*<0.01.

^a^ Before selection bias correction (unmatched sample).

^b^ After selection bias correction (matched sample).

^c^ Percentage increase was used to determine the output differential between members and non-members.

^d^ *t* test was conducted to determine if the predicted output of members are statistically different from non-members.

Finally, in Table 8, we present the heterogeneity impact of agricultural cooperative membership on TE based on farm size. Farm size is one of the most important scale indicators in the context of maize farmers in Nigeria [28]. The results show that cooperative members exhibit higher mean TE score than non-member across all the farm size groups. The results also show that there is no wide differences in percentage increase in mean TE obtained between members and non-members across different land size quantiles. The percentage increase in mean TE levels across the farm size groups ranges from 19.00% to 20.96%, suggesting that cooperative membership enhances TE of smallholders and poor farmers, as well as farmers with larger farm size in a fairly similar fashion.

**Table 8:** TE levels by farm size quantiles after bias correction.

SPF Model	Members	Non-members	% increase in TE ^a^	Test of means ^b^
*Farm size (ha)*				
1^st^ Quartile	0.75	0.62	20.96%	4.68***
2^nd^ Quartile	0.74	0.62	19.4%	3.01***
3^rd^ Quartile	0.75	0.63	19.0%	2.96***
4^th^ Quartile	0.75	0.63	20.0%	4.33***

Notes

*** *p*<0.01.

^a^ Percentage increase was used to determine the output differential between members and non-members.

^b^ *t*-test was conducted to determine if the predicted output of members is statistically different from non-members.

## 6. Conclusions and policy implications

The formation of agricultural cooperatives is a well promoted agricultural development policy initiative to help smallholder farmers cope with multiple production and marketing challenges. Using a nationally representative dataset of maize farmers in rural Nigeria, this study combines impact assessment technique with a parametric production frontier approach to investigate the impact of agricultural cooperative membership in enhancing TE. Specifically, we combined PSM and Greene [22]’s sample selection corrected SPF techniques to address selection biases stemming from observed and unobserved factors. We employed a stochastic meta-frontier technique to evaluate productivity differences between members and non-members. Our findings showed that the presence of selection bias cannot be rejected, thereby justifying the need to address biases stemming from unobservable attributes. Our analyses of the impact of cooperative membership show that there exist significant productivity differences between members and non-members. In particular, on average, the results of the MTE scores for members and non-members, respectively, are 0.61 and 0.41, implying that, cooperative membership favours efficient use of resources thereby making members more productive than non-members. This suggests that strategic policy attempts to enhance agricultural development as well as value chain development should provide continued incentives and encouragements for farmers to form as well as participate in agricultural cooperatives. Our estimates of the heterogeneity impacts of agricultural cooperatives suggest that membership enhances TE in a fairly similar fashion regardless of the scale of production. This further provides evidence that well-structured cooperatives could be an important policy tool for enhancing inclusive economic growth in developing countries, as evidenced in Wossen et al. [4] and Verhofstadt and Maertens [12].

Finally, our empirical results revealed the important roles of several farm managerial, socio-economic, and plot-specific factors play on farmers’ decision to participate in agricultural cooperatives. In specific, factors such as farmer’s age, household size, education, asset value, access to extension services, distance to seed market, intercropping, and drought experience are significant positive driving factors of farmers participation in cooperatives. In designing incentives for smallholder farmers to join agricultural cooperatives, policymakers should consider these factors to ensure that farmers can maximise the benefits of agricultural cooperatives. As an example, provision of a training programme designed to enlightening farmers on the benefits of membership, complemented with effective extension services to deliver technical capacity building in terms of the use of productive inputs and other management practices, as well as access to credit facilities which are key for enhancing technical efficiencies.

## Supporting information

S1 Appendix (DOCX)
