# Supplementary material for: Does agricultural cooperative membership impact technical efficiency of maize production in Nigeria: An analysis correcting for biases from observed and unobserved attributes
Source: PLoS One. 2021 Jan 22;16(1):e0245426. doi: 10.1371/journal.pone.0245426 (PMC7822389; doi:10.1371/journal.pone.0245426)
Supplement: S1 Appendix — (DOCX) [file pone.0245426.s001.docx]

**APPENDIX**

S1 Table. First stage estimates for addressing potential endogeneity

| Variables | Access to extension services | |  | Access to credit | |
| --- | --- | --- | --- | --- | --- |
|  | Coefficient | Std. Error |  | Coefficient | Std. Error |
| Gender | 0.009 | (0.121) |  | -0.054 | (0.110) |
| Age | 0.017 | (0.017) |  | -0.030** | (0.015) |
| Age squared | -0.000 | (0.000) |  | 0.000* | (0.000) |
| House size | 0.021 | (0.014) |  | 0.050*** | (0.012) |
| Education | 0.006 | (0.007) |  | 0.011* | (0.006) |
| Owned land | 0.015 | (0.107) |  | -0.096 | (0.095) |
| Log of asset value | 0.038 | (0.024) |  | 0.032 | (0.021) |
| Irrigation | 0.143 | (0.109) |  | -0.052 | (0.108) |
| Farm size | -0.023** | (0.012) |  | 0.002 | (0.011) |
| Risk | -0.056 | (0.087) |  | -0.041 | (0.084) |
| Access to extension |  |  |  | 0.307*** | (0.102) |
| Television/radio coverage for information | 0.135*** | (0.048) |  |  |  |
| Access to credit | 0.281*** | (0.095) |  |  |  |
| Awareness of credit sources |  |  |  | 0.148** | (0.045) |
| Distance to seed market | -0.001 | (0.003) |  | 0.004 | (0.003) |
| Row planting | 0.024 | (0.101) |  | -0.074 | (0.091) |
| Soil and water conservation | 0.064 | (0.073) |  | -0.030 | (0.070) |
| Inter cropping | 0.067 | (0.073) |  | 0.113 | (0.069) |
| Good soil | 0.021 | (0.082) |  | -0.120 | (0.077) |
| Drought | 0.217** | (0.087) |  | 0.082 | (0.086) |
| North central | -0.199* | (0.105) |  | 0.647*** | (0.096) |
| North west | -0.309*** | (0.109) |  | -0.190* | (0.109) |
| North east | 0.251 | (0.165) |  | 0.125 | (0.169) |
| South south | -0.618*** | (0.236) |  | -0.016 | (0.185) |
| South east | -0.490** | (0.236) |  | -0.821*** | (0.314) |
| Constant | -2.406*** | (0.538) |  | -1.090** | (0.451) |
| Observations | 2,228 |  |  | 2,228 |  |

Robust standard errors in parentheses, *** p<0.01, ** p<0.05, * p<0.1
